# Comprehensive Transcriptomic and Metabolomic Analysis Revealed the Functional Differences in Pigeon Lactation between Male and Female during the Reproductive Cycle

**DOI:** 10.3390/ani14010075

**Published:** 2023-12-24

**Authors:** Yuting Fu, Yan Song, Danli Jiang, Jianqiu Pan, Wanyan Li, Xumeng Zhang, Wenbin Chen, Yunbo Tian, Xu Shen, Yunmao Huang

**Affiliations:** 1 Guangdong Laboratory for Lingnan Modern Agriculture, Guangzhou 510225, China; fuyuting@zhku.edu.cn (Y.F.); songyanriver@163.com (Y.S.); danli0222@163.com (D.J.); panjianqiu@zhku.edu.cn (J.P.); lwanyan88@126.com (W.L.); zhangxumeng@zhku.edu.cn (X.Z.); 15874821125@139.com (W.C.); tyunbo@126.com (Y.T.); 2 College of Animal Science & Technology, Zhongkai University of Agriculture and Engineering, Guangzhou 510225, China; 3 Waterfowl Healthy Breeding Engineering Research Center, Guangdong Higher Education Institute, Guangzhou 510225, China

**Keywords:** pigeon, crop milk formation, metabolome, regulatory network, transcriptome

## Abstract

**Simple Summary:**

Lactation is an intriguing reproductive behavior in pigeons, both male and female pigeons participate in crop milk secretion and contribute to the care of their offspring through the division of labor. The question of whether the metabolism and formation of pigeon milk in both males and females follow the same mechanism is an interesting scientific question. We integrated lactation-associated metabolomic and transcriptomic data from the crop tissues of male and female pigeons during the reproductive cycles. We identified a total of 1413 metabolites across 18 crop tissues. During the reproductive cycles, the concentrations of estrone, L-ergothioneine, and L-histidine exhibited the most dynamic changes in females, while estrone, L-anserine, 1-methylhistidine, homovanillate, oxidized glutathione, and reducing glutathione showed the most dynamic changes in males. Through screening DAMs and DEGs, our findings revealed gender-specific differences in the metabolome and gene expressions, with several metabolites and pathways showing significant variations between males and females. Our findings shed light on the distinct modulation of pigeon crop milk metabolism between males and females and provide insights into the potential functions of male and female pigeon milk in the growth, development, and immunity of young pigeons.

**Abstract:**

Lactation is a unique reproductive behavior in pigeons, with the crop serving as the organ responsible for secreting pigeon milk. Both male and female pigeons can produce crop milk and rear their offspring through a division of labor. Since the time of the secretion of pigeon crop milk is different in the process of feeding the young, whether the metabolism and formation of pigeon milk use the same mechanism is a very interesting scientific question. However, the metabolic dynamics and underlying genetic mechanisms involved in the formation of pigeon crop milk remain unclear, particularly during the incubation–feeding reproductive cycle. In this study, we integrated lactation-associated metabolism and transcriptome data from the crop tissues of both male and female pigeons during the brooding and feeding stages. We mapped the changes in metabolites related to milk formation in the crop tissues during these stages. Through metabolome profiling, we identified 1413 metabolites among 18 crop tissues. During the breeding cycles, the concentrations of estrone, L-ergothioneine, and L-histidine exhibited the most dynamic changes in females. In contrast, estrone, L-anserine, 1-methylhistidine, homovanillate, oxidized glutathione, and reducing glutathione showed the most dynamic changes in males. Gender-specific differences were observed in the metabolome, with several metabolites significantly differing between males and females, many of which were correlated with cytokine binding, immunity, and cytochrome P450 activity. Using this dataset, we constructed complex regulatory networks, enabling us to identify important metabolites and key genes involved in regulating the formation of pigeon milk in male and female pigeons, respectively. Additionally, we investigated gender-associated differences in the crop metabolites of pigeons. Our study revealed differences in the modulation of pigeon crop milk metabolism between males and females and shed light on the potential functions of male and female pigeon milk in the growth, development, and immunity of young pigeons, an area that has not been previously explored. In conclusion, our results provide new insights into the metabolic regulation of pigeon crop milk formation during the brooding and breeding stages. Furthermore, our findings lay the foundation for the accurate development of artificial pigeon milk.

## 1. Background

Pigeons, an animal belonging to the genus Pigeon in the family Columbiformes, originated from the protopigeon species distributed in coastal areas and were the first birds to be domesticated by human beings [1]. Over time, pigeon breeding has become prevalent in countries worldwide. In the process of breeding, pigeons possess unique localization functions and physiological characteristics, making them an excellent research model. Previous studies have focused on various aspects of pigeons, such as their navigation mechanism [2], their use as a biomonitoring model for environmental pollution [3], their appearance traits [4], and their relevance as a pathological study model [5]. Numerous studies have been given more attention to the physiological characteristics of pigeon breeding [6,7,8].

A notable observation is that pigeons exhibit a distinctive reproductive pattern, where a pair of parent pigeons produces one or two eggs in a breeding cycle under natural conditions. As late adults, young pigeons rely on the full pulpy secretion provided by their parents until they are 28 days old, after which they start feeding independently [9,10,11]. This secretion, known as “pigeon milk”, is produced every 4 h by the crop of the parent pigeons and is regurgitated to feed the squabs. Pigeon crop milk plays a crucial role in the growth and development of young pigeons due to its high protein and fat content, although it contains low levels of carbohydrates [12]. Interestingly, the secretion of pigeon milk starts to decrease on the third day after the birth of the pigeons in females and on the thirteenth day after the birth of the pigeons in males [11,13,14,15]. It is now known that pigeon milk has growth-promoting properties [1], a regulatory intestinal microenvironment [16], and possesses immunological effects on the young [17].

Unlike mammals, both male and female pigeons produce pigeon crop milk [18], but they exhibit differences in lactation initiation times and durations. Actually, previous studies have demonstrated distinct metabolic characteristics during lactation between male and female pigeons [19], suggesting different mechanisms of milk production. Although previous studies have mainly focused on the formation mechanisms of pigeon crop milk, the chemical composition of pigeon crop milk and the underlying mechanisms affecting its production between male and female pigeons remain poorly understood. The formation of pigeon crop milk is strictly controlled by a complex regulatory mechanism. Growing evidence shows the synthesis of crop milk is controlled by prolactin- and insulin-activated pathways [20,21,22,23], which have an effect on the protein synthesis and apoptosis of crop epidermal cells [24]. Recently, a multiomics analysis identified some transcriptional regulatory elements that play key roles in the formation mechanisms of pigeon crop milk [25]. However, little literature is available on the chemical composition of pigeon crop milk and the underlying mechanism affecting its production between male and female pigeons.

Transcriptome sequencing is a powerful tool that provides insights into the complexity of gene expression in an organism. Metabolomics, as a downstream component of systems biology, can directly represent biological phenotypes. The integrated study of transcriptomics and metabolomics allows for a better understanding of biological phenomena. The physiological process of pigeon crop milk secretion from the crop tissue is strictly regulated through transcriptional and metabolic changes. In this study, we employed RNA-seq sequencing and a liquid chromatography–tandem mass spectrometry (LC/MS-MS) analysis to investigate the differences in lactation mechanisms between male and female pigeons during the breeding cycle. This approach provided a new perspective for elucidating the regulatory mechanisms of pigeon milk production.

## 2. Material and Methods

### 2.1. Sample Collection

Every three pairs of European meat pigeons of the Mimas strain (*Columba livia*) were randomly selected at three different stages: the PA stage indicates the incubation stage, the pigeons were on their seventh day of brooding; the PB stage indicates the rearing period, the pigeons were on their second day after the birth of the young; and the PC stage indicates the stage of overlapping incubation and rearing, where the breeding pigeons were simultaneously rearing the young from the first clutch until the twenty-second day while incubating a second clutch of eggs. The pigeon specimens were obtained from the breeding flock of Meizhou Jinlv Modern Agriculture Development Co., Ltd., (Meizhou, China). Each breeding pair was provided with the same food, mineral supplements, and water source. The environmental conditions were maintained at a temperature of 25–30 °C and a long photoperiod of 16 h of light, followed by 8 h of darkness. Blood samples were collected from the left-wing vein and the pigeons were euthanized before the immediate collection of crop tissue samples.

### 2.2. Morphological Changes and Histology of Crop Tissue

A portion of the crop samples was flash-frozen in liquid nitrogen and stored at −80 °C for a subsequent transcriptome and metabolome analysis. The remaining samples were fixed in 4% paraformaldehyde fixative for histological staining. The crop tissue sections were stained with oil red to assess pigeon development, while HE-stained sections were prepared for the examination of gonad development. Crop tissue sections stained with oil red were visualized and analyzed using Caseviewer 2.4 software (3DHISTECH, Budapest, Hungary) at a magnification of 4 × 10×. The three to five longest and thickest folds of crop tissues in each section were separately measured using Image-Pro Plus 6.0 analysis software.

### 2.3. RNA-Seq Process

Frozen tissues from each pigeon were individually used for RNA extraction and library construction, and RNA-seq detection and analysis were conducted using the BGISEQ-500 platform (BGI, Shenzhen, China). The raw sequencing data underwent filtering to remove low-quality reads, splice contamination, and reads containing unknown bases (“N”). The resulting clean reads were then aligned to the original pigeon reference genome using hierarchical indexing for spliced alignment of transcripts (HISAT) v2.1.0. Gene expression levels were quantified as fragments per kilobase of transcript per million mapped reads (FPKMs). A differential gene expression analysis between different sample groups was performed using DEGseq software (http://bioinfo.au.tsinghua.edu.cn/software/degseq/). Genes with a fold change greater than two and a *p*-value adjusted to q ≤ 0.001 were considered differentially expressed genes (DEGs). The functional annotation of genes was performed using Blast2GO and KOBAS (http://kobas.cbi.pku.edu.cn, accessed on 15 July 2022) for Gene Ontology (GO) and the Kyoto Encyclopedia of Genes and Genomes (KEGG) analysis. The GO and KEGG enrichment analyses were carried out using the phyper function in R software (v4.2.2) with the false discovery rate (FDR) correction applied to the *p*-values. Functions with a q-value of ≤0.05 were considered significantly enriched. A bioinformatics analysis was conducted using the Dr. Tom web platform provided by BGI (http://report.bgi.com, accessed on 8 May 2022).

### 2.4. Metabolite Detection and Data Analysis

Parental pigeon crop samples were thawed at 4 °C after being removed from storage in a −80 °C refrigerator. Tissue samples weighing 25 mg were mixed with 800 μL of a methanol–water (1:1) solution and 3 mm steel beads (average diameter) in each sample. The samples were homogenized by shaking at 60 Hz for 5 min using a tissue grinder (JXFSTPRP, Shanghai, China). Subsequently, the samples were centrifuged at 25,000× *g* for 10 min at 4 °C. To ensure the reproducibility and system stability of the LC–MS/MS analysis, 200 μL of supernatant from each sample was combined to create a mixed-quality control (QC) sample. The remaining supernatant from each sample was separated and freeze-dried. Finally, the dried supernatant samples were redissolved in deionized water and subjected to a mass spectrometry analysis. Metabolite separation and detection were performed using a Waters UPLC I-Class Plus system (Waters, Milford, MA, USA) coupled with a Q Exactive high-resolution mass spectrometer (Thermo Fisher Scientific, Waltham, MA, USA). The analysis was conducted in both positive and negative ionization modes using electrospray ionization. The LC–MS experiments were carried out by BGI (Shenzhen, China). The downstream data obtained from the mass spectra were imported into Compound Discoverer 3.2 software (Thermo Fisher Scientific, Waltham, MA, USA) and matched with the UW Metabolome Database (BMDB), mzCloud database, and ChemSpider online database for the metabolite annotation and data analysis. Quality control (QC) samples were included to assess the reproducibility of the results obtained by pooling all the samples. A principal component analysis (PCA) was performed to visualize metabolic alterations between groups. Metabolites showing a fold change of ≥1.2 or ≤0.83 and a *p*-value < 0.05 were considered differential metabolites (DAMs). The metabolite annotation and pathway analysis were conducted using the KEGG database.

### 2.5. qRT-PCR Qualification

A total of 5 differentially expressed genes (DEGs) were selected for the qPCR analysis to validate the RNA-seq data. The total RNA was extracted from crop tissues using Trizol (Thermo Fisher Scientific, Waltham, MA, USA), followed by reverse transcription using TAKARA reagents(Takara Bio, Beijing, China). The levels of target mRNA were then quantified using SYBR Priemix Ex Taq (Thermo Fisher Scientific, Waltham, MA, USA) in a quantitative real-time PCR (qRT-PCR) experiment. Each sample was analyzed in triplicate and the primer sequences used for qRT-PCR are provided in Table S1.

### 2.6. Statistical Analysis

A statistical analysis was conducted using GraphPad Prism 8.0.2 software (GraphPad Software, Boston, MA, USA) to compare the data in a statistically significant manner. A metabolomic data analysis was performed using the MetaboAnalyst website. Student’s *t*-test was employed for all statistical analyses. The results were presented as means ± standard errors (SEMs) and the statistical significance was set at *p* < 0.05.

## 3. Results

### 3.1. Morphological and Structural Changes in the Crop of Male and Female Pigeons across Different Breeding Cycles

**Figure 1:**
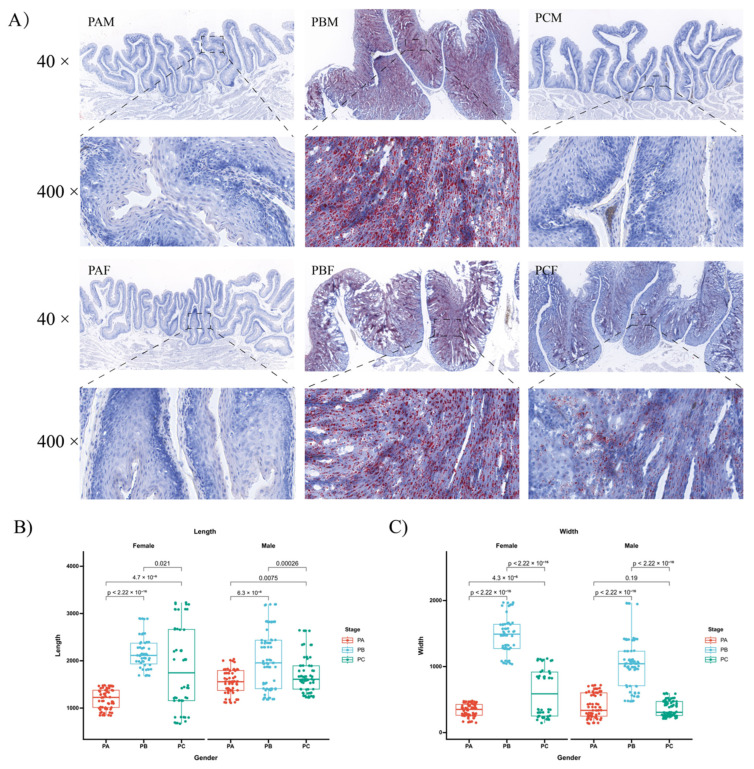
Changes in the crop of male and female pigeons in different breeding cycles. (**A**) Oil-red-stained sections of the crop collected from male and female pigeons at different stages: PA, PB, and PC. PAM, PBM, and PCM represent male pigeons in PA, PB, and PC, respectively, while PAF, PBF, and PCF indicate female pigeons in PA, PB, and PC, respectively. (**B**) The length of crop folds during the three periods. (**C**) Crop-fold widths during the three periods. The values were expressed as mean ± standard error (SEM) (n = 3). The significance level is set at *p* < 0.05 for each comparison.

Oil-red-stained frozen sections were prepared from the same region to investigate changes in the crop during the reproductive cycle. Lipids appeared in red and lipid droplets accumulated strongly and were clearly present in the PB stage. A rapid decrease in the number of stained cells and the size of lipid droplets was observed in the PC stage, although not to the extent observed in the PA stage (Figure 1A). Throughout the reproductive cycle, significant morphological changes were observed in the crop tissues. In both sexes, the crop folds initially elongated and thickened, with adjacent folds merging and initiating lipid secretion. Subsequently, there was a gradual decrease in lipid secretion, resulting in thinner folds until they returned to their original state. Importantly, the crops of the PCM and PCF groups exhibited distinct morphological variations and differences in oil red staining intensity (Figure 1B,C).

### 3.2. Metabolite Analysis Showed Differential Metabolite Accumulation during Breeding Cycles

For the metabolite analysis, a total of 18 samples were included, obtained from nine pairs of pigeons across three reproductive stages. Each reproductive stage had three biological replicates for both male and female pigeons. The obtained metabolite profiles underwent a principal component analysis (PCA) with classification based on gender (Figure S1). The results revealed a relative separation of the three different collection groups at different periods in the score plot. A total of 3813 metabolites were detected, of which 1413 were known and 2400 were unidentified (Supplementary File S1), with lipids (23.37%), amino acids, peptides, and analogues (16.09%), and carbohydrates (6.9%) representing the top three classifications of metabolites. By applying the screening criteria of fold change ≥1.2 or ≤0.83 and *p* < 0.05 to all detected metabolites in each comparison group, a total of 498 differential accumulation metabolites (DAMs) were identified (Table S2 and Supplementary File S2). By comparing the levels of differential accumulation metabolites (DAMs) in the parents across different reproductive cycles, we observed that the two comparison groups (PCF vs. PAF and PAM vs. PBM) exhibited a higher number of DAMs compared to the other comparison groups. This finding suggested significant changes in the physiological and metabolic activities of the female pigeon during the PC and PA stages. In male pigeons, the most pronounced alterations in physiological and metabolic activities were primarily observed in the PA and PB stages.

During the incubation and rearing periods, a total of 26 differential metabolites were identified between PAF and PBF, including an increase in L-ergothioneine, emetine, and L-histidine, and a decrease in 2-amino-2-deoxy-4-O-β-D-galactopyranosyl-D-glucose, oleate, and 9-fluoro-5β-androstane-3,11,17-trione in the crop tissues. Furthermore, a total of 272 differential metabolites were identified between PAM and PBM, with a decrease in hispidulin, carnosine, and 2-quinolinecarboxylic acid in the crop. Between PBF and PCF, a total of 18 differential metabolites were identified, including a decrease in L-isoleucine and L-ergothioneine, and an increase in adenine in the PC stage. Similarly, a total of 82 metabolites were identified between PBM and PCM, with increased concentrations of L-anserine, 1-methyl-L-histidine, L-glutathione oxidized, and L-glutathione reduced in the PC stage. Between PAF and PCF, a total of 92 differential metabolites were identified, with decreased levels of estrone and 5,6-dihydrouracil, and an increased level of L-histidine in the PC stage. Additionally, a total of 117 metabolites were identified between PAM and PCM, with increased concentrations of homovanillate, 3β-androstanediol, and Υ-aminobutyric acid, while decreased levels of L-anserine, prostaglandin E2, and 1-methyl-L-histidine were observed in the PC stage. Across the three stages, the concentration of 3β-androstanediol gradually increased in females while declining in males (Supplement File S2).

The metabolic pathway enrichment analysis of differential metabolites was conducted using the KEGG database to gain further insights into the biological mechanisms associated with lactation in parental pigeons. The top 9–10 enriched KEGG pathways for each comparison group were selected and presented in charts based on a statistical significance criterion of *p* < 0.05 (Supplement File S3). Notably, female pigeons exhibited more pronounced differences in metabolites involved in lipid metabolism in the crop tissues, including fatty acid biosynthesis, pantothenic acid, and CoA biosynthesis, and unsaturated fatty acid biosynthesis. On the other hand, male pigeons showed distinct variations in glutathione metabolism, ferroptosis, and neuroactive ligand–receptor interactions.

### 3.3. Transcriptome Analysis Reveals the Differentially Expressed Genes during the Breeding Cycles

A transcriptome analysis was conducted to identify differentially expressed genes (DEGs) in the crop of parental pigeons during different breeding cycles. Each sample produced an average of 6.37 Gb of data, with an average matching rate of 81.34% for sample-matched genomes and 68.38% for matched gene sets. Additionally, 641 new genes were predicted and a total of 15,491 expressed genes were detected, including 14,858 known genes and 633 being predicted as new genes. Furthermore, 14,177 new transcripts were identified, of which 13,536 belonged to known protein-coding genes, while 641 were transcripts of newly discovered protein-coding genes. DEGs were identified across the six groups based on the screening criteria of *p* < 0.05 and FC > 2 (Table S3 and Supplement File S4). Among all the comparison groups, the PCF vs. PAF and PAM vs. PBM groups exhibited the highest accumulation of DEGs, indicating that transcriptome changes primarily occurred during the PC stage for females and the PB phase for males throughout the breeding cycle.

**Figure 2:**
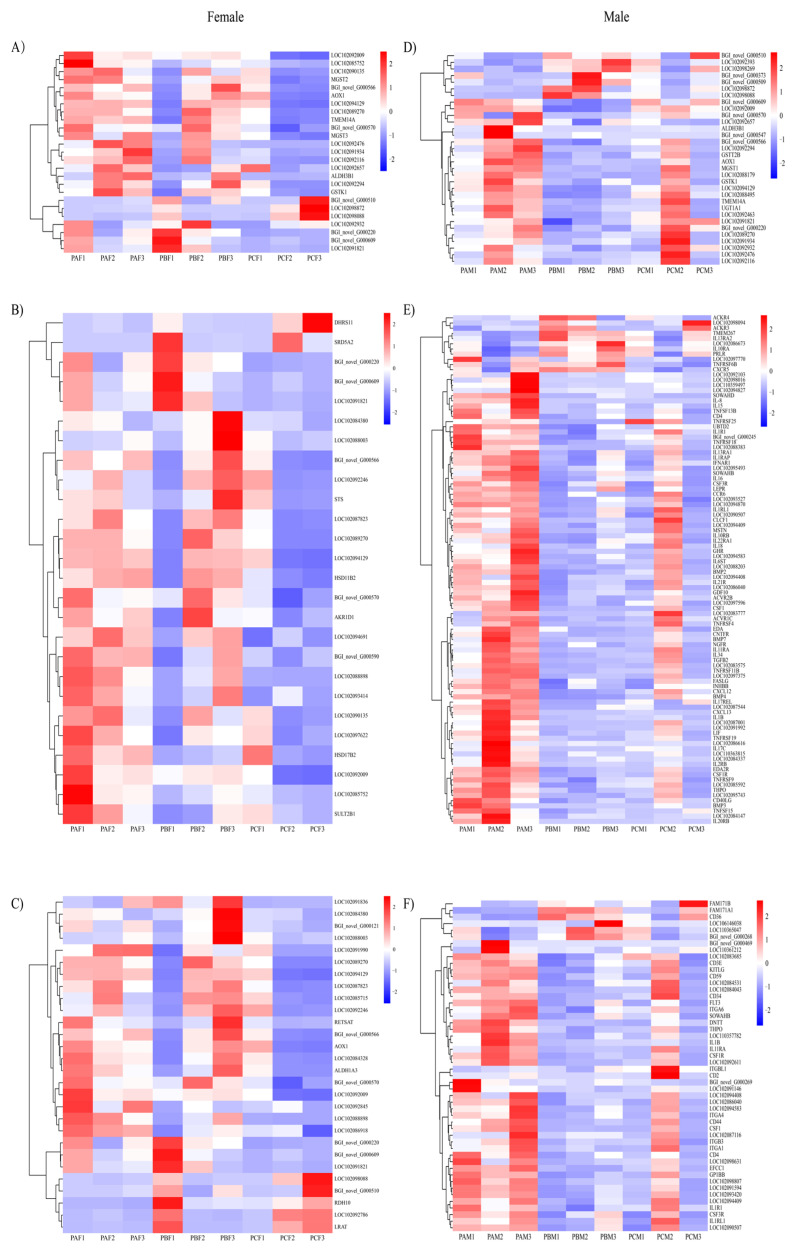
Heat map of the KEGG pathway TOP3 enriched by differentially expressed genes in the crop of male and female pigeons during different reproductive cycles. Female pigeons (**A**) drug metabolism—cytochrome P450, (**B**) steroid hormone biosynthesis, and (**C**) retinol metabolism. Male pigeons (**D**) drug metabolism—cytochrome P450, (**E**) cytokine-cytokine receptor interactions, and (**F**) hematopoietic cell lineage.

The KEGG analysis of the DEGs among the different comparison groups provided insights into the enrichment of genes in differential biological pathways. Based on statistical significance criteria adjusted for multiple testing (adjusted *p* value < 0.05), significantly enriched pathways were identified (Supplement File S5). Among these pathways, females exhibited a significant enrichment of DEGs primarily in PPAR signaling pathways, while males demonstrated enrichment in immune-related pathways, such as the IgA-producing intestinal immune network, the differentiation of Th1 and Th2 cells, and the formation of neutrophil extracellular traps. These findings were consistent with the results obtained from the metabolomic analysis. Additionally, male pigeons showed an enrichment in calcium signaling pathways and osteoclast differentiation. The KEGG pathways enriched in the top three were screened according to *p* values from groups of female and male pigeons at different times, respectively. Heat maps of the associated differential genes were shown (Figure 2). The top three KEGG pathways in females were drug metabolism—cytochrome P450, retinol metabolism, and steroid hormone biosynthesis. In males, the top three pathways were drug metabolism—cytochrome P450, cytokine–cytokine receptor interactions, and hematopoietic cell lineage.

### 3.4. Functional Gene Involvement in Lactation

**Figure 3:**
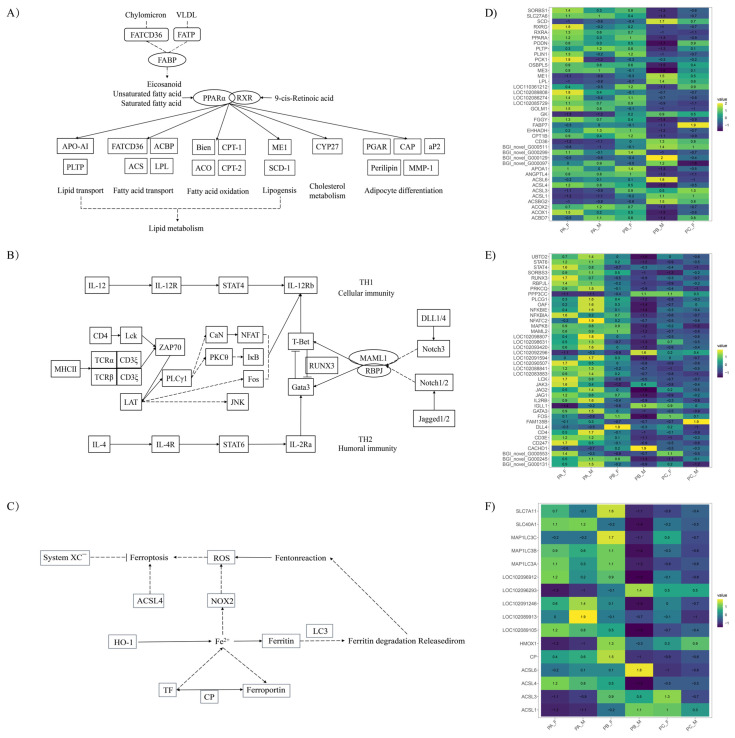
Significantly different KEGG pathways during the breeding cycle in the crop of male and female pigeons. (**A**) PPAR signaling pathway, (**B**) TH1 and TH2 cell differentiation pathway, and (**C**) ferroptosis were differentially expressed in the crop of parental pigeons, while (**D**–**F**) were their corresponding differential gene expression heatmaps, respectively.

By integrating the enriched KEGG pathways in both differentially expressed genes and differential abundance metabolites across all comparison groups, we identified a significant expression difference in genes related to lipid metabolism and immune pathways between male and female pigeons during the reproductive cycle. These findings highlighted the essential role of pigeon milk secreted by males in providing enhanced nutritional support for offspring growth and development throughout the breeding cycle. Notably, we specifically focused on the PPAR signaling pathway and TH1 and TH2 cell differentiation pathways to identify statistically significant gene differences, elucidating the variations in gene expression between the parental birds (Figure 3).

### 3.5. Combined Analysis of Metabolome and Transcriptome in Parent Pigeons

**Figure 4:**
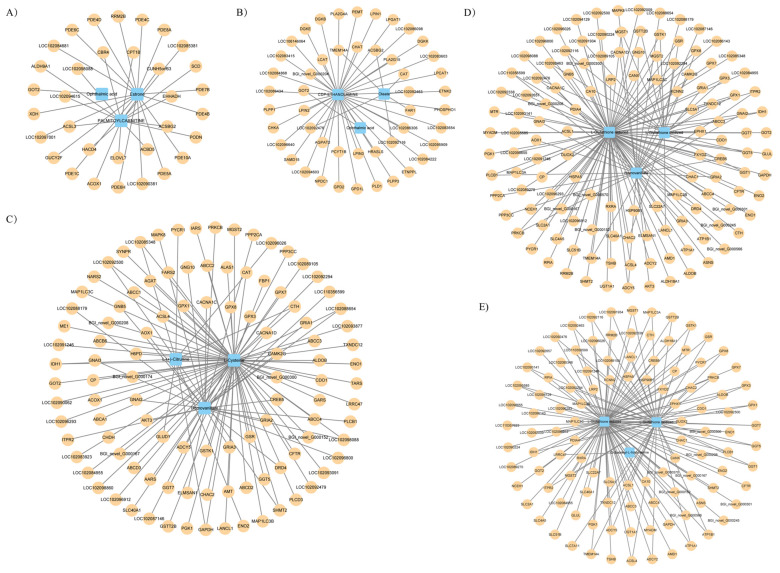
The transcript–metabolite correlation network represents the top 10 DAMs and their corresponding DEGs involved in the crop of male and female parent pigeons across the different breeding cycles. (**A**) PAF vs. PCF, (**B**) PBF vs. PAF, (**C**) PAM vs. PCM, (**D**) PBM vs. PAM, and (**E**) PCM vs. PBM. Blue quadrangles denote DAMs, yellow circles denote DEGs, and the linking lines were determined by Spearman’s < −0.6 or >0.6, respectively (q < 0.05).

To further explore the relationship between gene expression and metabolite accumulation in parental pigeons during different breeding cycles, correlation tests and co-biological annotations were performed on female and male comparisons. In all comparison groups, we selected the top 10 differential metabolites and their corresponding differential genes and used Spearman’s correlation analysis to calculate the correlation and produce Cytoscape plots. It can be seen that there were more differential metabolites in the crop of male pigeons at different times and a greater number of associated differential genes involved compared to females (Figure 4). In the comparison of PAF vs. PCF, the differential metabolites in the crop of female pigeons were estrone, ophthalmic acid, and palmitoylcarnitine, while in the comparison of PBF vs. PAF, the differential metabolites were CDP-ethanolamine, oleate, and ophthalmic acid. In male pigeons, the differential metabolite networks across the three comparison groups consisted of oxidation glutathione, reduction glutathione, and homovanillate, which is the main metabolite of dopamine.

### 3.6. Gender-Specific Differences in the Metabolome and Gene Expression of Crop Tissues

The significantly different compounds are shown with a fold-change threshold >1.20 or <0.83 and a *p*-value < 0.05 in all comparisons (Table S2). Males and females exhibited distinct levels of metabolites, with 66 DAMs identified in PA, 64 DAMs identified in PB, and 44 DAMs identified in PC. The differentially abundant metabolites for each comparison were presented (Table 1 and Supplementary File S2). In the PA stage, females displayed a high concentration of glyceraldehyde 3-phosphate and a low level of gamma-glutamyl-gamma-glutamyl-S-methylcysteine compared to males. In the PB stage, females exhibited a high concentration of 5′-S-methyl-5′-thioinosine and a low level of reduced glutathione compared to males. Lastly, in the PC stage, females demonstrated a high concentration of 3β-androstanediol and a low level of cystathionine compared to males.

Furthermore, we evaluated the differences in gene expression associated with lactation between genders in the crop tissue. The complete list of DEGs is provided in Supplementary File S4, with the most pronounced expression differences observed in the PB stage. Compared to PBF, PBM showed an upregulation of 448 genes and a downregulation of 3068 genes. These genes were significantly enriched in 20 pathways (Table 2 and Supplementary File S5). The most significant signaling pathways associated with lactation between males and females in the PB stage included the metabolism of xenobiotics by cytochrome P450, calcium signaling pathway, axon guidance, retinol metabolism, PPAR signaling pathway, and MAPK signaling pathway, among others. In the PA stage, DEGs between males and females were significantly enriched in signaling pathways related to cytokine–cytokine receptor interactions, neuroactive ligand–receptor interactions, and phagosome. Meanwhile, in the PC stage, DEGs between males and females were significantly enriched in signaling pathways related to drug metabolism—cytochrome P450; steroid hormone biosynthesis; drug metabolism—other enzymes; cardiac muscle contraction, metabolism of xenobiotics by cytochrome P450, neuroactive ligand–receptor interactions, retinol metabolism, calcium signaling pathway, and linoleic acid metabolism.

### 3.7. Validation of Candidate DEGs by Quantitative Real-Time PCR

To verify the accuracy of our RNA-Seq sequencing results, we selected 14 key DEGs from the key pathways identified and performed a quantitative real-time PCR (qRT-PCR) expression analysis in male and female parents of different breeding cycles. All gene expression patterns selected using qRT-PCR were similar to RNA-Seq, confirming the reliability of RNA-Seq data and the subsequent analysis (Figure S2).

## 4. Discussion

The morphology of the pigeon crop undergoes variations during different periods of the breeding cycle [26]. In response to prolactin, the pigeon crop begins to proliferate during the incubation period and accumulates protein and lipid. Eventually, the keratinized epithelial cells collect a substantial amount of nutrients, degenerate, and shed off, forming pigeon milk [27]. Pigeon milk is known for its richness in protein and fat [1], with approximately 90% of the protein being casein and the remaining consisting of keratin and immunoglobulin, among others [28]. Early studies have shown that the fat content in pigeon milk primarily consists of neutral fat and phospholipids, with cholesterol, triglycerides, and free fatty acids as the main components [29]. The relative composition of pigeon milk secreted by pigeons varies at different times, particularly as the proportion of feed in pigeon milk gradually increases with the age of the offspring [1]. Regarding lipids, the lipid percentage in pigeon milk starts to decrease until the pigeons reach 10 days of age [29]. Thus, the lipid content and amino acid accumulation serve as indicators of whether male and female pigeons secrete pigeon milk. It is of practical significance to unveil the differences in lactation mechanisms between male and female pigeons during the reproductive cycle. While transcriptome and microRNAome analyses have shed some light on the mechanism of pigeon milk secretion, its metabolomics remains largely unexplored.

In previous studies, the production and secretion of pigeon milk were found to be regulated by several hormones, with prolactin playing a major role [30]. In the statistical results of transcriptomics, it was observed that only the expression level of the PRLR receptor among the hormone receptors changed at different times. This indicated that the crop primarily responded to prolactin (PRL) in the blood during different reproductive cycles, which was consistent with the findings of previous studies.

Lipids in pigeon milk primarily originate from extracellular transport [20]. Previous studies have suggested that peroxisome proliferator-activated receptors (PPARs) play important roles in the regulation of lipid, glucose, and energy homeostasis, as well as cell proliferation and differentiation [31]. PPARs exist in three isoforms, PPAR-α, PPAR-β/δ, and PPAR-γ [32], with PPAR-α and PPAR-γ being involved in fatty acid oxidation and adipogenesis [33]. The activation of PPAR-α has a significant impact on lipoprotein metabolism, lipid and cholesterol transport, and can reduce plasma triglyceride levels by increasing the activity of LPL (lipoprotein lipase) [34]. A high expression of the LPL gene has been observed in the lactating crop in previous studies [14], and it was also identified as a differential gene in this study. Studies have shown that the expression of the fatty acid transporter protein FAT/CD36 in pigeons is regulated by PPAR-γ [35]. However, in the present study, despite differences in FAT/CD36 gene expression at different periods, only PPAR-α exhibited differential expression in the parental crop, being upregulated at PAF and downregulated at PCF. On the other hand, the downstream genes of PPAR-γ, such as Gyk and PEPCK, were differentially expressed in different groups. It is generally believed that there is a time difference between females and males in preparing for lactation, with females initiating lactation earlier than males [20]. In the current experiment, the presence of oil red staining was observed in the PCF group, but not in the PCM group of the parent pigeons from the PC stage, which were in the late stage of incubation with offspring, further supporting this view.

In mammals, the colostrum primarily consists of IgA and is believed to play an immunological role in intestinal protection [36]. It has been observed that IgA is present in the serum of 1-day-old pigeons and also serves a local protective function in their intestines [37]. As the pigeon crop is a glandular tissue [26], it is hypothesized that immune substances in pigeon milk are produced by T and B cells that migrate into the crop from the bloodstream. The KEGG pathway associated with IgA production was found to be significantly enriched only in male pigeons. This suggests that male pigeons may possess a greater concentration of immune substances in their pigeon milk to protect their offspring.

TH1 cells are primarily responsible for mediating cellular immune responses, while TH2 cells mediate humoral immunity. These two types of cells work together in relative balance to maintain overall body health [38]. Naive CD4 T cells recognize and bind antigens presented by MHC II molecules on the surface of lymphoid dendritic cells, subsequently activating the STAT or SMAD pathway to differentiate into various effector cell subsets [39]. The JAK/STAT pathway, located upstream of the PI3K/AKT pathway, also plays a role in promoting keratinocyte proliferation. In this study, it was found that IL-12 induced Th1 cell differentiation and activated STAT4, whereas IL-4 induced Th2 cell differentiation and activated STAT6. The differentiation of CD4+ T cells into Th1 and Th2 effector cells was regulated by transcription factors T-bet and GATA3, respectively. The overexpression of T-bet promoted differentiation into the Th1 lineage, while the deletion of Gata3 prevented differentiation into the Th2 lineage. Moreover, the overexpression of Gata3 in Th1 cells converted their polarity to a Th2 phenotype [40]. However, fluctuations in Gata3 expression throughout the reproductive cycle were only observed in males.

The expression of genes related to antioxidant activity was found to be upregulated in the crop of parental pigeons during the carrier period [27]. In the metabolic group analyzed in this study, the identified differential metabolites were oxidized glutathione and reduced glutathione and, similarly, the differential metabolic pathway included glutathione metabolism, which overlapped with the ferroptosis pathway. Glutathione peroxidase 4 (GPX4) is the fourth member of the selenium-containing GPX family, and in mammals, only GPX4 exhibits the ability to scavenge membrane lipid hydrogen peroxide products [41], which is one of the indicators for determining cellular ferroptosis [42]. Although GPX4 itself did not show a differential expression between the different groups, other enzymes of the GPX family, such as GPX1, GPX3, GPX7, and GPX8, were considered.

In our results, significant differences in metabolites involved in the glutathione metabolic pathway, such as L-glutathione oxidized and L-glutathione reduced, were observed. These metabolites were found to be upregulated in the PAM vs. PBM comparison and downregulated in the PBM vs. PCM comparison. Correspondingly, GPX1 was upregulated in the PAM vs. PBM comparison, while GPX3, GPX7, and GPX8 were downregulated. In the PBM vs. PCM comparison, GPX1 and GPX7 showed no significant changes, whereas GPX3 and GPX8 were upregulated. Interestingly, in the PCM vs. PAM comparison, where there were no differences in metabolites, GPX1 levels were downregulated, while GPX7 levels were upregulated. Similarly, in the PCF vs. PAF comparison, where there were no metabolite differences, only GPX1 levels were downregulated.

The acyl-CoA synthetase long-chain family member 4 (ACSL4) is known for its ability to preferentially activate long-chain polyunsaturated fatty acids and to play a crucial role in driving ferroptosis [43]. The inhibition of ACSL4 expression may be a key mechanism that allows cells to avoid ferroptosis, and this regulation could be influenced by cell–cell and cell–extracellular matrix adhesion signaling pathways [44,45]. Interestingly, these pathways were also found to be enriched during parental pigeon lactation [27]. Studies on crop lactation have revealed that the rapidly proliferating germinal layer cells experience hypoxia due to an inadequate blood supply [27], and ASCL4 expression is upregulated in ischemic tissues, thereby positively regulating ferroptosis [46].

Both parental pigeons possess the ability to produce crop milk, but the differences in the composition of crop milk between male and female pigeons are rarely examined. Increasingly more evidence is showing that the milk composition and synthesis vary significantly with sexual effects [47,48]. Lactating in crop tissues can lead to an increase in the expression of antioxidant protein-encoding genes [27]. In the PB stage, crop tissues in males exhibited a higher concentration of reduced glutathione than in the females. Glutathione acts as an antioxidant [49]. Reduced glutathione has been demonstrated to play a key role in controlling the cellular immune response [50]. These findings highlight the crucial role of high glutathione concentrations in the crop of lactating male pigeons, enhancing the immune function of pigeon milk.

## 5. Conclusions

In this study, we conducted a comprehensive metabolomic and transcriptomic analysis to examine the variations in the lipid and amino acid secretion of pigeon milk between male and female pigeons during different reproductive cycles. Through the screening of metabolites and candidate genes associated with lipid metabolism and immunity, we identified and discussed the differences in lactation mechanisms between male and female pigeons. Our findings enhance our understanding of the divergent mechanisms and roles of pigeon milk secretion in male and female pigeons.

## Figures and Tables

**Table 1:** The top 5 differentially abundant metabolites in the crop tissue of male and female pigeons.

Comparison	Name	Fold Change	*p*-Value	Molecular Weight
PAM vs. PAF	Glyceraldehyde 3-phosphate	7.04	0.02	169.99835
	N-Acetyl-L-glutamic acid	0.12	0.02	189.064
	Homovanillate	0.10	0.03	204.04016
	1-Arachidoyl-sn-glycero-3-phosphate	0.04	0.04	466.30605
	Gamma-glutamyl-gamma-glutamyl-S-methylcysteine	0.03	0.04	393.12053
PBM vs. PBF	5′-S-Methyl-5′-thioinosine	20.95	0.01	298.07362
	L-Cystine	19.16	0.03	240.02379
	Carnosine	0.10	0.03	226.10663
	Sedoheptulose 1,7-bisphosphate	0.09	0.03	370.00547
	Reduced glutathione	0.04	0.04	307.08385
PCM vs. PCF	3β-Androstanediol	7.66	0.00	292.24022
	Spermidine	5.18	0.02	145.15816
	Dehydrosiphonochalynol	0.56	0.03	302.16639
	L-cystathionine	0.38	0.04	222.0674
	Cystathionine	0.23	0.01	222.06738

**Table 2:** Gender-specific differences in pathway enrichment in crop tissues.

Comparison	Kegg_Term	Enrich_Ratio	*p*-Value	q-Value
PAF vs. PAM	Viral protein interaction with cytokine and cytokine receptor	1.88 × 10^−1^	3.14 × 10^−6^	6.56 × 10^−4^
	Cytokine–cytokine receptor interaction	9.09 × 10^−2^	4.32 × 10^−4^	3.07 × 10^−2^
	Neuroactive ligand–receptor interaction	7.56 × 10^−2^	5.60 × 10^−4^	3.07 × 10^−2^
	Phagosome	9.88 × 10^−2^	5.8 × 10^−4^	3.07 × 10^−2^
PBF vs. PBM	Drug metabolism—cytochrome P450	6.19 × 10^−1^	5.03 × 10^−8^	1.30 × 10^−5^
	Metabolism of xenobiotics by cytochrome P450	5.74 × 10^−1^	2.50 × 10^−7^	3.22 × 10^−5^
	Calcium signaling pathway	3.34 × 10^−1^	1.4 × 10^−6^	1.27 × 10^−4^
	Axon guidance	3.55 × 10^−1^	4.46 × 10^−6^	2.88 × 10^−4^
	Retinol metabolism	4.50 × 10^−1^	9.87 × 10^−5^	3.64 × 10^−3^
	PPAR signaling pathway	4.12 × 10^−1^	9.46 × 10^−5^	3.64 × 10^−3^
	MAPK signaling pathway	3.16 × 10^−1^	9.80 × 10^−5^	3.64 × 10^−3^
	Sphingolipid metabolism	4.35 × 10^−1^	1.94 × 10^−4^	6.17 × 10^−3^
	Drug metabolism—other enzymes	4.13 × 10^−1^	2.15 × 10^−4^	6.17 × 10^−3^
	Vascular smooth muscle contraction	3.48 × 10^−1^	3.21 × 10^−4^	8.27 × 10^−3^
	Cell adhesion molecules	3.50 × 10^−1^	4.78 × 10^−4^	1.12 × 10^−2^
	Cholesterol metabolism	4.09 × 10^−1^	6.54 × 10^−4^	1.41 × 10^−2^
	Mucin type O-glycan biosynthesis	4.72 × 10^−1^	9.75 × 10^−4^	1.68 × 10^−2^
	Arachidonic acid metabolism	4.06 × 10^−1^	9.27 × 10^−4^	1.68 × 10^−2^
	Renin secretion	3.90 × 10^−1^	8.87 × 10^−4^	1.68 × 10^−2^
	Hippo signaling pathway	3.20 × 10^−1^	1.05 × 10^−3^	1.70 × 10^−2^
	Porphyrin metabolism	4.59 × 10^−1^	1.43 × 10^−3^	2.13 × 10^−2^
	Cytokine–cytokine receptor interaction	3.12 × 10^−1^	1.49 × 10^−3^	2.13 × 10^−2^
	Steroid hormone biosynthesis	4.15 × 10^−1^	1.61 × 10^−3^	2.18 × 10^−2^
	cAMP signaling pathway	3.04 × 10^−1^	2.38 × 10^−3^	3.08 × 10^−2^
PCF vs. PCM	Drug metabolism—cytochrome P450	3.10 × 10^−1^	3.57 × 10^−6^	8.41 × 10^−4^
	Steroid hormone biosynthesis	2.64 × 10^−1^	1.19 × 10^−5^	1.40 × 10^−3^
	Drug metabolism—other enzymes	2.13 × 10^−1^	5.24 × 10^−5^	3.09 × 10^−3^
	Cardiac muscle contraction	1.56 × 10^−1^	3.96 × 10^−5^	3.09 × 10^−3^
	Metabolism of xenobiotics by cytochrome P450	2.55 × 10^−1^	7.21 × 10^−5^	3.24 × 10^−3^
	Neuroactive ligand–receptor interaction	1.22 × 10^−1^	8.25 × 10^−5^	3.24 × 10^−3^
	Retinol metabolism	2.17 × 10^−1^	2.22 × 10^−4^	7.49 × 10^−3^
	Calcium signaling pathway	1.14 × 10^−1^	1.54 × 10^−3^	4.03 × 10^−2^
	Linoleic acid metabolism	2.35 × 10^−1^	2.06 × 10^−3^	4.87 × 10^−2^

## Data Availability

The data sets supporting the results of this article were included within the article and additional files. Raw sequencing reads are available at Genome Sequence Archive (GSA) database of the National Geoscience Data Centre (NGDC) (bio-project accession: PRJCA021856, RNA-seq accessions CRA013803).

## Supplementary Materials

The following supporting information can be downloaded at: https://www.mdpi.com/article/10.3390/ani14010075/s1, Figure S1. PCA analysis of metabolites in pigeon crop tissues in both males and females. (A) PCA analysis of metabolites in pigeon females. (B) PCA analysis of metabolites in male pigeons. Figure S2. qPCR validation during the breeding cycle in the crop of male and female pigeons. Table S1. Primer used for qPCR validation. Table S2. Statistics of different accumulated metabolites in the crop of male and female pigeons. Table S3. Statistics of different differential genes in the crop of male and female pigeons. Supplementary File S1. All metabolites identified across the pigeon reproductive cycles. Supplementary File S2. Differential accumulation metabolites identified across the pigeon reproductive cycles. Supplementary File S3. Metabolic pathway enrichment analysis of differential metabolites across the pigeon reproductive cycles. Supplementary File S4. Differential expression genes identified across the pigeon reproductive cycles. Supplementary File S5. Pathway enrichment analysis of differential expression genes across the pigeon reproductive cycles.
