# Supplementary material for: Comprehensive Transcriptomic and Metabolomic Analysis Revealed the Functional Differences in Pigeon Lactation between Male and Female during the Reproductive Cycle
Source: Animals (Basel). 2023 Dec 24;14(1):75. doi: 10.3390/ani14010075 (PMC10778231; doi:10.3390/ani14010075)
Supplement: Supplementary file 1 [file animals-14-00075-s001.zip › Table S1 Primer used for qPCR validation.docx]

| Genes | Primer sequence(5’-3’) | Length (bp) |
| --- | --- | --- |
| PRLR | GTGAAACCGCACCACTTAGC | 272 |
|  | TGTACTGACTGCCCCACAAG |  |
|  | CAAAATCCCCGCACAGTCAA |  |
| ACSL3 | GCGCAGCAATAGTTCATGGG | 140 |
|  | TGGTTTGCCATCCACCGTAA |  |
| MOGAT1 | GAGGAAGCAAGTGAGGCGAT | 192 |
|  | AGCACAGCTTTCCACACACT |  |
| PRKCQ | CCCGACTTTCTGTGAGCACT | 225 |
|  | AGAGGTCCATCCCTGGAGAC |  |
| LOC102089571 | TGCCGGTGAATGATACCGAG | 246 |
|  | TGGTCTCACCATTGGGCTTC |  |

Primer used for qPCR validation
