# Supplementary material for: Comprehensive Transcriptomic and Metabolomic Analysis Revealed the Functional Differences in Pigeon Lactation between Male and Female during the Reproductive Cycle
Source: Animals (Basel). 2023 Dec 24;14(1):75. doi: 10.3390/ani14010075 (PMC10778231; doi:10.3390/ani14010075)
Supplement: Supplementary file 1 [file animals-14-00075-s001.zip › Table S2 Statistics of different accumulated metabolites in the crop of male and female pigeons.docx]

Statistics of different accumulated metabolites in the crop of male and female pigeons

| Comparison groups^1^ | DAMs number | Up-regulated DAMs number | Down-regulated DAMs number |
| --- | --- | --- | --- |
| PAF vs PBF | 26 | 17 | 9 |
| PBF vs PCF | 18 | 12 | 6 |
| PCF vs PAF | 92 | 42 | 50 |
| PAM vs PBM | 272 | 121 | 151 |
| PBM vs PCM | 82 | 62 | 20 |
| PCM vs PAM | 117 | 70 | 47 |
| PAM vs PAF  PBM vs PBF  PCM vs PCF | 66  64  44 | 20  28  18 | 46  36  26 |

Note: F indicated Female, M indicated Male
