# Supplementary material for: Comprehensive Transcriptomic and Metabolomic Analysis Revealed the Functional Differences in Pigeon Lactation between Male and Female during the Reproductive Cycle
Source: Animals (Basel). 2023 Dec 24;14(1):75. doi: 10.3390/ani14010075 (PMC10778231; doi:10.3390/ani14010075)
Supplement: Supplementary file 1 [file animals-14-00075-s001.zip › Table S3 Statistics of different differential genes in the crop of male and female pigeons.docx]

Statistics of different differential genes in the crop of male and female pigeons

| Comparison groups | Total DEGs | Up-regulated DEGs | Down-regulated DEGs |
| --- | --- | --- | --- |
| PAF vs PBF | 884 | 546 | 338 |
| PBF vs PCF | 1289 | 302 | 987 |
| PCF vs PAF | 2551 | 1682 | 869 |
| PAM vs PBM | 3982 | 628 | 3354 |
| PBM vs PCM | 1695 | 1418 | 277 |
| PCM vs PAM  PAM vs PAF  PBM vs PBF  PCM vs PCF | 1862  618  3516  1026 | 1494  349  448  350 | 368  269  3068  676 |
