# Supplementary figures and images for: Comprehensive Transcriptomic and Metabolomic Analysis Revealed the Functional Differences in Pigeon Lactation between Male and Female during the Reproductive Cycle
Source: Animals (Basel). 2023 Dec 24;14(1):75. doi: 10.3390/ani14010075 (PMC10778231; doi:10.3390/ani14010075)

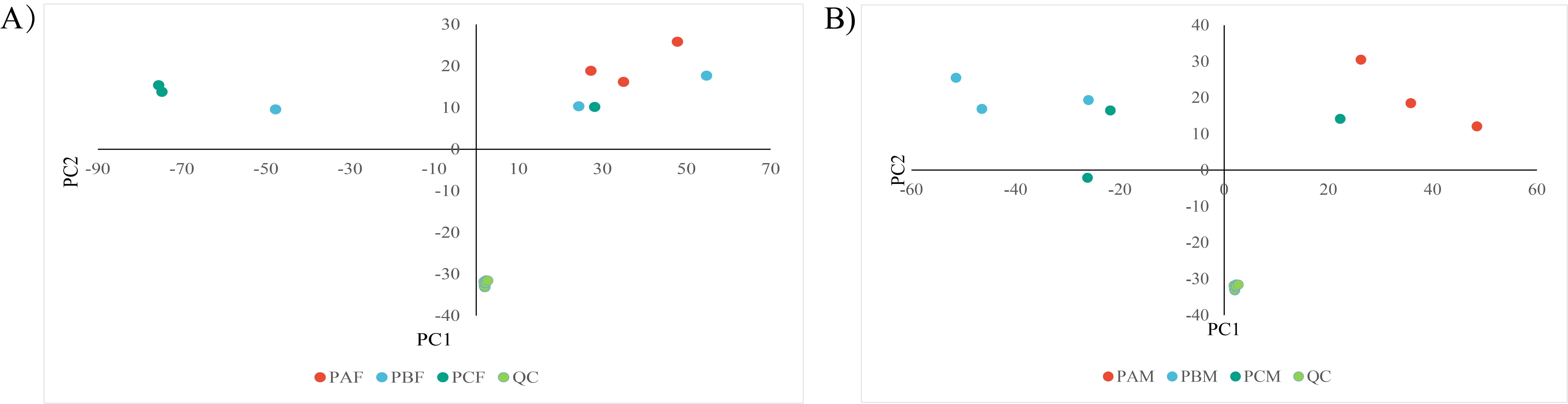

Supplement: Supplementary file 1 [file animals-14-00075-s001.zip › Figure S1 PCA analysis of metabolites in pigeon crop tissues in both males and females .tif]

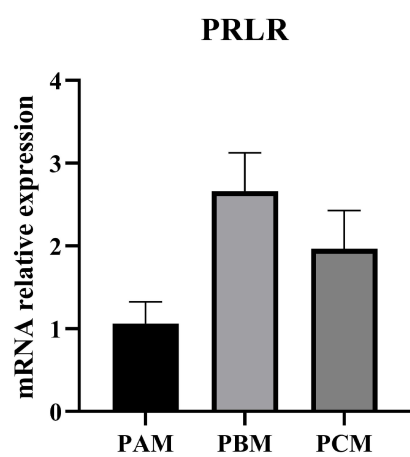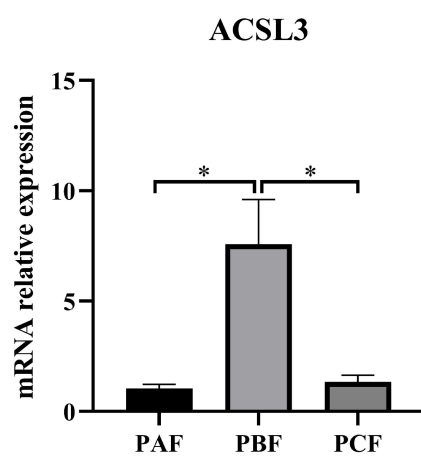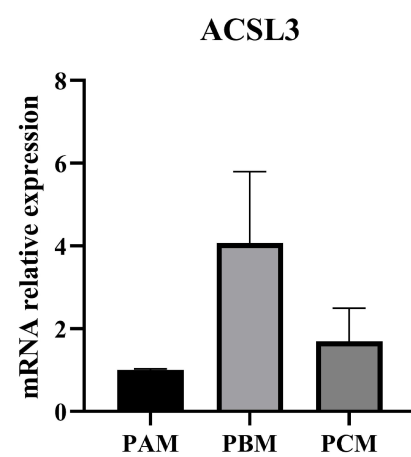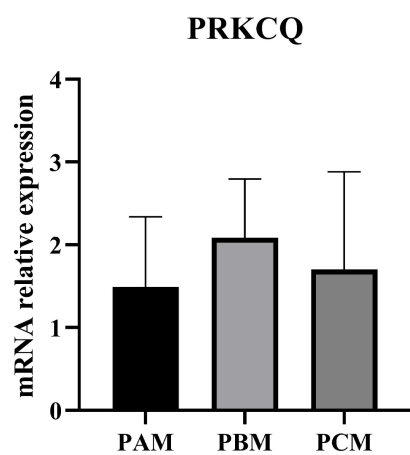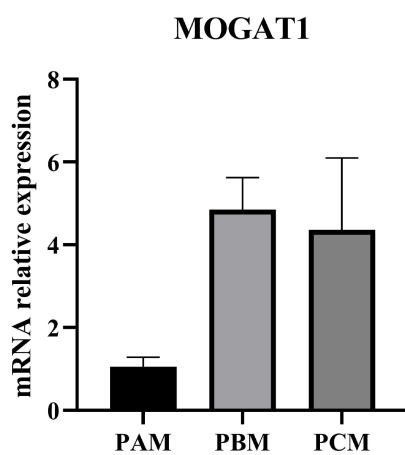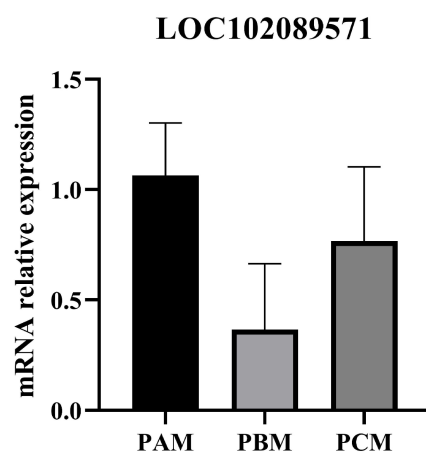

Supplement: Supplementary file 1 [file animals-14-00075-s001.zip › Figure S2 qPCR validation during the breeding cycle in the crop of male and female pigeons.pdf]
